# Effects of AMPK/PGC-1α on gluconeogenesis in skeletal muscle of animals under overwintering starvation and its molecular mechanism: review

**DOI:** 10.5713/ab.25.0459

**Published:** 2025-10-22

**Authors:** Chaoyong Huang, Huiyu Cheng, Binhong Wen, Wei Li, Baohua Luo, Dubala Wu, Jingshun Wang, Sile Hu, Jianghong Wu

**Affiliations:** 1 College of Animal Science and Technology, Inner Mongolia Minzu University, Tongliao, China; 2 Hulunbuir Agricultural and Animal Husbandry Science Research Institute, Hulunbuir, China; 3 College of Life Science and Food, Inner Mongolia Minzu University, Tongliao, China

**Keywords:** AMP-activated Protein Kinase, Forkhead Box O1, Overwintering Starvation Stress, Peroxisome Proliferator-activated Receptor Gamma Coactivator 1-alpha, Pyruvate Dehydrogenase Kinase 4, Skeletal Muscle Gluconeogenesis

## Abstract

During the wintering period characterized by feed scarcity, animals activate the sympathetic nervous system (SNS)-β-adrenergic receptor (βAR)-AMP-activated protein kinase (AMPK)/peroxisome proliferator-activated receptor gamma coactivator 1-alpha (PGC-1α) signaling pathway in their bodies. This activation increases the transcriptional activity of *forkhead box O1* (*FOXO1*) transcription factor and *pyruvate dehydrogenase kinase 4* (*PDK4*), leading to the upregulation of gluconeogenesis-limiting genes *phosphoenolpyruvate carboxykinase* (*PEPCK*) and *glucose-6-phosphatase* (*G6Pase*). Additionally, it enhances the functions of carnitine palmitoyltransferase 1 (CPT1), uncoupling protein 1 (UCP1), cluster of differentiation 36 (CD36), and fatty acid transport protein (FATP). This promotes promote intermuscular fat oxidation and the production of gluconeogenesis precursor glycerol, thereby enhancing animal skeletal muscle gluconeogenesis to maintain animal energy homeostasis and life activities under low temperature and starvation conditions. This article describes the effects of overwintering starvation stress on the AMPK/PGC-1α signaling pathway and its molecular mechanism in animal skeletal muscle gluconeogenesis. The aim to provide a theoretical basis for optimizing sustainable breeding strategies and improving animal production performance in alpine regions.

## INTRODUCTION

During the winter, animals face the dual stress of low temperature and feed shortage. To adapt to the insufficient energy supply of the body, animals hibernate or store energy in the form of fat before wintering, and maintain normal life activities of the body through precise regulation of gluconeogenesis, hormone regulation, and energy metabolism-related pathways. Hibernating animals actively decrease their body temperature and metabolism rate to reduce energy consumption and prolong the utilization of limited energy resources [1]. Non-hibernating animals mainly dependent on gluconeogenesis to satisfy their body energy supply, especially ruminants. Due to the particularity of their digestive system, the direct absorption of the glucose from diet is limited. Gluconeogenesis, as the key pathway for energy supply, plays an important role in promoting animals’ adaptation for low temperature stress and hunger [2]. As the main metabolic organ for glucose, skeletal muscle alleviates the body’s energy deficiency under winter starvation stress by regulating pathways such as gluconeogenesis and fatty acid oxidation [3,4]. Compared with the liver, skeletal muscle gluconeogenesis has unique metabolic adaptability. When energy supply is insufficient, activation of the sympathetic nervous system (SNS)-β-adrenergic receptor (βAR)-AMP-activated protein kinase (AMPK)/peroxisome proliferator-activated receptor gamma coactivator 1-alpha (PGC-1α) metabolic pathway in skeletal muscle can compensate for the deficiency of liver gluconeogenesis.

Under cold and starvation conditions, animals’ sensing of their body’s energy status is enhanced, inducing the activation of energy-sensitive metabolic regulatory factors such as *AMPK*, upregulating *PGC-1α* expression, and promoting the transcriptional expression of key rate-limiting genes for gluconeogenesis including *phosphoenolpyruvate carboxykinase* (*PEPCK*) and *glucose-6-phosphatase* (*G6Pase*), thereby enhancing the body’s gluconeogenesis and improving the animal’s cold tolerance [5,6]. In addition, winter starvation stress induces activation in the animal’s SNS, stimulating the sympathetic nerve endings and adrenal medulla to secrete norepinephrine and β-adrenaline, and promoting the binding of these catecholamines to receptors, activating the AMPK/PGC-1α signaling pathway, enhancing intermuscular fat mobilization and the production of glycerol, a precursor of gluconeogenesis, in the muscle, to ensure the body’s energy homeostasis [7,8]. In addition, under conditions of long-term nutrient deprivation, varius non-coding RNAs such as long noncoding RNA (lncRNA) NBR2 are key regulators in maintaining the body’s sustained energy supply by modulating the activity of core energy metabolism factors such as AMPK, thereby influencing their downstream signaling pathways [9]. Currently, there is a lack of systematic reviews on how animals adapt to insufficient exogenous energy supply through adaptive metabolic pathways under winter starvation stress. This review elucidates the regulatory mechanism of AMPK/PGC-1α on animal skeletal muscle gluconeogenesis under overwintering starvation stress and the differences in gluconeogenesis between monogastric and ruminant animals. It aims to reveal the molecular mechanism of energy metabolism in cold-season grazing sheep and the adaptive strategies of energy deficiency in different species, in order to provide a theoretical basis for the precise regulation of mutton quality and sustainable breeding strategies and feeding management in high-altitude regions.

## REGULATORY MECHANISM OF AMPK/PGC-1α UNDER WINTER STARVATION STRESS

### The sympathetic nervous system-β-adrenergic receptor signaling pathway

Ruminants have a compound stomach structure and fermentation system that can utilize endogenous energy replenishment pathways. When responding to winter starvation stress, they mainly activate the SNS-βAR-AMPK/PGC-1α pathway in the body, regulate the interaction between β-adrenaline and AMPK pathways, enhance fatty acid oxidation, glucose metabolism, and gluconeogenesis functions, and maintain energy balance. When energy is scarce, the SNS is activated, stimulating the sympathetic nerve endings and adrenal medulla to release catecholamine hormones such as norepinephrine and epinephrine, which then bind to βAR, activate the cAMP/protein kinase A (PKA) signaling cascade in the cell, and enhance energy metabolism [10]. At the same time, low temperature stimulation can also activate the SNS through the skin temperature receptors, induce the release of norepinephrine and its binding to βAR, and then activate the stimulatory G protein (Gs), prompting its Gsα subunit to bind to GTP to form a Gsα-GTP active complex, which can directly bind to and activate adenylate cyclase (AC), catalyzing the conversion of ATP to cAMP, and significantly increasing the intracellular cAMP level [11]. As a second messenger, cAMP binds to the regulatory subunit of PKA and activates the catalytic subunit of PKA to regulate downstream pathways such as AMPK and PGC-1α [12,13]. Studies have shown that cold exposure activates the SNS, leading to the release of noradrenaline, which acts on βAR, significantly increases PGC-1α protein levels and *forkhead box O1* (*FOXO1*) mRNA expression, upregulates PEPCK, G6Pase, and carnitine palmitoyltransferase 1 (CPT1) level, promotes gluconeogenesis and fatty acid oxidation, and maintains the body’s energy homeostasis [14]. In addition, cold induces SNS-βAR to activate PKA activity. Activated PKA phosphorylates the Ser428 site of liver kinase B1 (LKB1), promoting the formation of a complex between LKB1 and the cascade reaction regulatory protein/MO25 protein, and translocates from the nucleus to the cytoplasm. LKB1, as an upstream kinase of AMPK, directly phosphorylates the Thr172 site of the AMPKα subunit, enhancing its catalytic activity [15,16]. Studies have shown that under long-term chronic cold exposure or the action of β3-adrenergic agonists, the levels of *AMPKα1* mRNA and protein in mice are significantly increased, phosphorylating the Ser79 site of acetyl-CoA carboxylase (ACC), reducing the production of malonyl-CoA, alleviating the inhibitory effect of malonyl-CoA on CPT1, and enhancing hormone-sensitive lipase (HSL), promoting fatty acid oxidation and the production of gluconeogenic precursor glycerol, thereby maintaining the body’s energy homeostasis under long-term cold conditions [17].

Under starvation conditions, a decrease in plasma glucose levels in mice induces glucagon secretion and binding to the G protein-coupled receptor (GPCR) on the liver cell membrane, leading to conformational changes in the GPCR and activation of the coupled Gαs protein, thereby activating AC and increasing intracellular cAMP levels. Increased cAMP activates PKA and causes its catalytic subunit to enter the nucleus and phosphorylate the cAMP response element-binding protein (CREB) Ser133 site and the CREB regulated transcription coactivator 2 (CRTC2) coactivator, triggering CREB activation and CRTC2 nuclear translocation to form a CREB-CRTC2 transcription complex, which can specifically bind to the CRE site of the *PGC-1α* gene promoter, activate the transcription of *PGC-1α*, thereby enhancing the expression of its downstream genes, *PEPCK* and *G6PC*, and ultimately enhancing the gluconeogenesis capacity of mice [18]. In addition, the activated PKA catalytic subunit directly phosphorylates the Ser133 site of the CREB protein to induce its conformational change, thereby recruiting its co-activator histone acetyltransferase CBP/p300, catalyzing histone H3/H4 acetylation, inducing CRE chromatin remodeling, facilitating the binding of CREB to target gene promoters such as *PEPCK* and *G6Pase*, and enhancing the body’s gluconeogenesis [19]. At the same time, phosphorylated CREB forms a complex with the coactivator CREB-regulated transcriptional coactivator 2 and histone acetyltransferase CBP/p300, promoting hypomethylation and hyperacetylation of the chromatin H3K27 site, targeting the cAMP response element (CRE) in the promoter region of the *PGC-1α* gene, and activating the transcription of *PGC-1α* [20,21]. After being activated by the calcium signaling pathway, CREB can also directly bind to the conserved site of the *PGC-1α* promoter. PGC-1α, acting as a coactivator, directly binds MEF2 to enhance its transcriptional activity, and MEF2 can in turn bind to the MEF2 site in the PGC-1α target transcription factor region to maintain *PGC-1α* expression, and PGC-1α also binds to transcription factors such as FOXO1 and HNF4α to co-activate gluconeogenic enzyme genes (*PEPCK*, *G6Pase*), thereby promoting gluconeogenesis [22]. In addition, PKA also activates mitogen-activated protein kinase kinase 3/6, triggering the dual phosphorylation of p38 mitogen-activated protein kinase (p38 MAPK) at Thr180/Tyr182. p38 MAPK targets and promotes the phosphorylation of PGC-1α at Thr262/Ser265, relieving the inhibitory effect of the p160 MBP repressor protein on the *PGC-1α* transcriptional active region, exposing the *PGC-1α* transcriptional activation domain and enhancing its binding efficiency with the KIX domain of the Mediator complex subunit MED1, thereby inducing the activation of peroxisome proliferator-activated receptor alpha (PPARα), upregulating the expression of lipolysis genes such as *CPT1*, and promoting fatty acid beta-oxidation. PGC-1α also enhances the activity of the transcriptional activator FOXO1 of PEPCK and G6Pase, synergistically promoting the body’s gluconeogenesis and improving cellular adaptation to energy deficiency [23,24].

**Figure 1:**
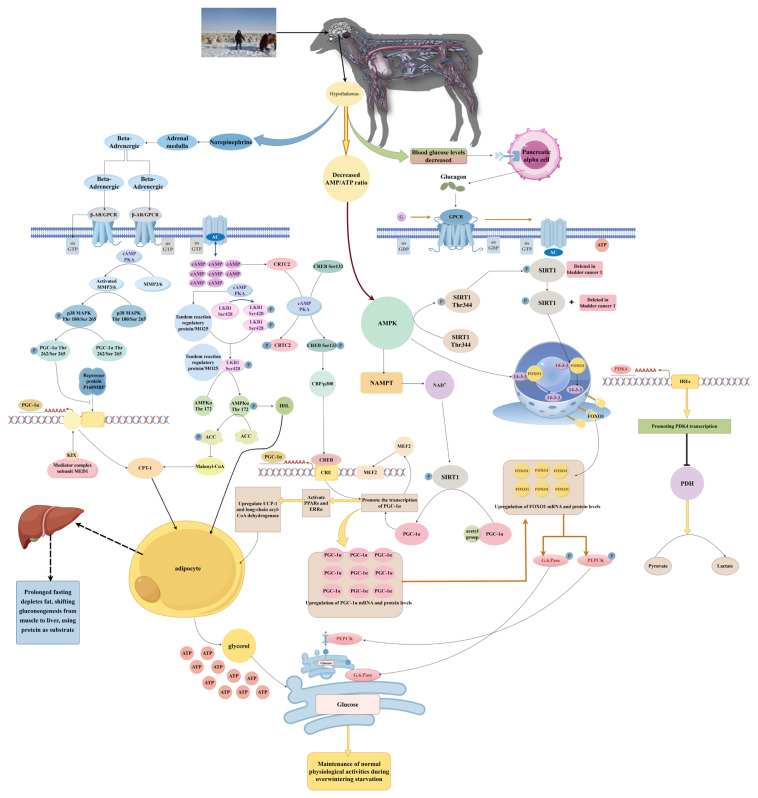
Molecular mechanism of AMPK/PGC-1α regulating gluconeogenesis in animal skeletal muscle during winter starvation. AMPK, AMP-activated protein kinase; PGC-1α, peroxisome proliferator-activated receptor gamma coactivator 1-alpha.

In addition to the aforementioned mechanisms, monogastric animals rely more on energy sensing, upregulating the AMPK pathway to enhance energy supply. During winter, starvation stress increases the AMP/ATP ratio, inducing phosphorylation of AMPK at Thr172, activating CREB, and promoting its binding to the CRE site in the promoter region of the *PGC-1α* gene. This promotes the expression of downstream fatty acid oxidation and gluconeogenesis genes, thereby increasing the animal’s cold tolerance [25,26]. In summary, ruminants, due to the endogenous energy compensation provided by ruminal fermentation, mainly induce the activation of βAR, initiate the Gs-AC-cAMP-PKA pathway, and then upregulate the expression of *AMPK* and *PGC-1α* by phosphorylating LKB1 and CREB/p38 MAPK, thereby enhancing the expression of genes such as *CPT1*, *PEPCK*, and *G6Pase*, and consequently enhancing fatty acid oxidation and gluconeogenesis. Monogastric animals rely more on the increase in the AMP/ATP ratio in response to energy deficiency, directly promoting the phosphorylation of AMPK Thr172 and activating PGC-1α, thereby upregulating genes related to fatty acid oxidation and gluconeogenesis to maintain energy homeostasis (Figure 1).

### Regulation by non-coding RNAs

Non-coding RNAs promote fat oxidation and gluconeogenesis in the body by activating the AMPK signaling pathway, thus maintaining whole-body energy homeostasis when feed is scarce. When animals are energy-deficient, miRNAs target and bind to AMPK negative regulatory factors or the 3’ untranslated region of *AMPK*, inhibiting its expression activity, thereby activating AMPK signaling, enhancing the body’s energy metabolism, and adapting to energy stress [27]. Studies have shown that when mice are fasting, the level of miR-22 in the body increases significantly, inhibiting the expression of *transcription factor 7* (*TCF7*), an effector molecule in the Wnt pathway, relieving its inhibitory effect on gluconeogenesis genes, and upregulating the expression of gluconeogenesis-limiting genes such as *PEPCK* and *G6PC*, thereby promoting glucose production [28]. Under cold conditions, miR-455 in mice targets and inhibits the translation of hypoxia-inducible factor 1α inhibitor, reduces AMPK hydroxylation, promotes its phosphorylation activation, upregulates the expression of *uncoupling protein 1* (*UCP1*) and *PGC-1α*, and enhances the body’s heat production capacity [29]. Unlike non-hibernating animals, miR-33 levels increase during hibernation in brown bears, directly targeting the 3’ untranslated region of the prkaa1 gene and inhibiting the catalytic subunit of AMPK encoded by the gene. This leads to the inhibition of AMPK phosphorylation of ACC and activation of GLUT4, which in turn leads to a decrease in fatty acid oxidation and glucose uptake in the body, thereby reducing energy consumption during the winter [30].

Under overwintering starvation stress, lncRNA promotes metabolic adaptation of skeletal muscle by enhancing the expression of AMPK and its downstream metabolic factors. Under glucose starvation conditions, lncRNA NBR2 is significantly upregulated and interacts with the AMPKα subunit, inducing conformational rearrangement of AMPK, increasing the accessibility of its kinase domain to substrates and enhancing enzyme activity, promoting AMPK phosphorylation of ACC, inhibiting malonyl-CoA production, relieving inhibition of CPT1, and enhancing fatty acid oxidation to maintain the body’s energy supply [31]. Similarly, in lncTUG1-overexpressing mice, lncTUG1 can competitively bind to miR-204, alleviate the inhibitory effect of miR-204 on *SIRT1* mRNA 3’ UTR, activate the AMPK/ACC signaling pathway, upregulate the expression of fat mobilization genes such as *ATGL*, *PPARα*, *PGC-1α*, and *UCP1*, promote the body’s fat metabolism and the generation of gluconeogenic precursor glycerol, and enhance systemic energy metabolism [32]. Circular RNA (circRNA) also plays a key regulatory role in the post-transcriptional regulation of AMPK/PGC-1α. Similar to hibernating animals, under the condition of adequate nutrition, circPTPN4 is overexpressed, significantly reducing the protein expression level of *PGC-1α*, and relieving its inhibitory effect on the rate-limiting enzyme nicotinamide phosphotransferase (NAMPT) in NAD^+^ synthesis by sponge adsorption of miR-499-3p, thereby upregulating the NAD^+^/NADH ratio and inhibiting the activation of the AMPK signaling pathway to reduce the body’s catabolism. Under starvation conditions, NAD^+^ levels decrease, and AMPK will be strongly activated to respond to the energy crisis [33]. Studies have found that when organismal energy demand increases, circERI3 in the cytoplasm can directly bind to DNA damage binding protein 1, inhibit its ubiquitination and degradation, promote its binding to the *PGC-1α* promoter region, enhance *PGC-1α* transcriptional expression, promote glucose uptake and the production of gluconeogenic precursor lactate, accelerate mitochondrial oxidative phosphorylation, and provide energy support for cells [34]. In summary, non-coding RNA has a dual role in regulating AMPK. Under winter starvation, animals primarily enhance fatty acid oxidative phosphorylation and the generation of gluconeogenic precursor lactate by regulating the expression of AMPK/PGC-1α and its downstream factors *UCP1*, *PEPCK*, and *G6PC*. At the same time, when the body has excess energy, it inhibits AMPK/PGC-1α expression to ensure the animal’s adaptability in energy deficiency and maintain its normal energy metabolism in stressful environments such as low temperature or starvation.

## REGULATION OF THE AMPK PATHWAY AND PGC-1α EXPRESSION UNDER WINTER STARVATION STRESS

Under overwintering starvation stress, AMPK regulates the expression and activity of *PGC-1α* by directly phosphorylating PGC-1α or indirectly mediating the deacetylation of Sirtuin 1 (SIRT1), coordinating metabolic adaptation under short-term energy stress and long-term starvation. Under energy stress, AMPK in C2C12 muscle cells changes the spatial conformation of PGC-1α by directly phosphorylating the Thr177 and Ser538 sites of PGC-1α, facilitating its binding to nuclear transcription factors or other coactivators, thereby enhancing the transcriptional coactivation activity of *PGC-1α* [35]. Under cold stimulation, the expression of *PGC-1α* in the gastrocnemius muscle of mice is significantly upregulated. PGC-1α activates ERRα to directly bind to the NR site of the promoter region of *pyruvate dehydrogenase kinase 4* (*PDK4*), inducing the transcriptional expression of *PDK4*. PDK4 then inhibits PDC through phosphorylation, reduces glucose oxidation, and promotes fatty acid oxidation, thereby providing normal metabolism of the body under short-term energy stress [36]. AMPK can also activate SIRT1 by increasing intracellular NAD^+^ levels, thereby regulating the expression and activity of PGC-1α. When energy is scarce, AMPK phosphorylates and inhibits ACC, reduces malonyl-CoA levels, promotes the entry of long-chain fatty acids into mitochondria for beta-oxidation, and enhances the body’s fatty acid oxidation. At the same time, it significantly increases *NAMPT* expression, catalyzes the conversion of nicotinamide to nicotinamide mononucleotide, and synthesizes SIRT1’s essential cofactor NAD^+^ through NMNAT enzyme, activating SIRT1 deacetylase activity, and then deacetylates PGC-1α, enhancing its metabolic activity [37]. AMPK also directly phosphorylates SIRT1 at Thr344, inducing dissociation of SIRT1 from the inhibitory factor bladder cancer protein 1 and enhancing SIRT1’s ability to deacetylate downstream target genes. Activated SIRT1 activates *PGC-1α* and its co-activating transcription factors, such as PPARs, ERRs, FOXO, and MEF2, through deacetylation, thereby upregulating the expression of fatty acid oxidation genes such as *UCP1* and long-chain fatty acyl-CoA dehydrogenase, as well as mitochondrial synthesis genes, ERRs, thereby increasing energy output to adapt to insufficient energy supply in animals [37,38]. It can be inferred from this that AMPK may continuously activate PGC-1α through the regulatory mechanism of the NAD^+^-SIRT1-PGC-1α axis, promote the body’s fat metabolism, and meet the organism’s energetic demands to adapt to the dilemma of long-term energy deficiency. In addition, when the adipose reserves of animals are exhausted during long-term hunger, the body switches from primarily relying on fat oxidation for energy to mobilizing protein as the main energy source to maintain normal life activities. Studies have shown that when the fat reserves in fish deplete to 0.7% to 5% of their total body weight, cortisol levels rise significantly, promoting protein mobilization in the liver, internal organs, and muscles, significantly increasing the level of glucogenic amino acids, and generating carbon skeletons through deamination, which enter the tricarboxylic acid cycle for oxidation and energy supply [39]. The residual amino acids, such as alanine and glutamate, are converted into glucose through the gluconeogenesis pathway, jointly maintaining blood glucose homeostasis under extreme starvation conditions.

## REGULATION OF SKELETAL MUSCLE GLUCONEOGENESIS BY AMPK/PGC-1α

Under overwintering starvation stress, AMPK/PGC-1α activates *FOXO1* transcriptional activity, upregulates *PEPCK* and *G6Pase* expression, and maintains the endogenous supply of glucose in skeletal muscle to meet energy needs. During energy deficiency, AMPK is activated as a cellular energy sensor, promoting the deacetylation and nuclear translocation of *FOXO1*, upregulating the expression of *PEPCK* and *G6Pase*, and enhancing gluconeogenesis to maintain normal life activities of the body [40]. In the fasting state, AMPK activates the NAD^+^-dependent deacetylase SIRT1 activity by promoting NAMPT expression and NAD^+^ biosynthesis, thereby deacetylating PGC-1α, enhancing the transcriptional activity of *PGC-1α*, and activating the expression of nuclear respiratory factors NRF-1/NRF-2 and mitochondrial transcription factor A (TFAM) to maintain energy homeostasis [41,42]. When energy is sufficient, insulin promotes phosphorylation of FOXO1 at Thr24 and Ser253, facilitating its binding to 14-3-3 protein, masking the DNA binding domain of FOXO1 in the nucleus, and preventing it from binding to the target gene promoter [43]. At the same time, in the fed state, the LKB1/AMPK signaling pathway can also phosphorylate class IIa HDACs, promote their binding to 14-3-3 and retention in the cytoplasm, inhibit the deacetylation of FOXO1, and reduce the transcriptional activity of gluconeogenic genes including *G6Pase* and *PEPCK* [44]. Under starvation conditions, AMPK phosphorylates the Ser22 site of FOXO1. After Ser22 phosphorylation, the negatively charged phosphate group is introduced, which hinders the binding of 14-3-3 protein to other sites on FOXO1 through electrostatic repulsion and allosteric effects, thereby relieving the 14-3-3-mediated extranuclear retention and transcriptional inhibition of FOXO1, thereby promoting the enrichment of FOXO1 in the cell nucleus, and enhancing its accessibility and binding ability to target DNA, ultimately potentiating its transcriptional activity [45]. In addition, PGC-1α, as a transcriptional coactivator of FOXO1, synergizes with FOXO1 to activate the expression of gluconeogenic genes such as *G6Pase* and *PEPCK* [46]. During fasting, glucagon activates the cAMP/PKA pathway, substantially upregulating *PGC-1α* mRNA and protein levels, promoting the binding of PGC-1α to the N-terminal domain of FOXO1. Co-expression significantly enhances FOXO1 transcriptional activity, thereby promoting the binding of PGC-1α, FOXO1 to the *G6Pase*/*PEPCK* promoter and potentiating gluconeogenesis [47].

AMPK also upregulates *PDK4* expression, promoting the flow of glucose metabolism intermediates such as pyruvate into the gluconeogenesis pathway, and enhancing intramuscular gluconeogenesis. Overactivation of AMPK in mouse myoblasts can promote the upregulation of the expression of the downstream target gene *PDK4* of *FOXO1* transcriptional regulation, leading to the inhibition of PDH and thus suppressing glucose oxidation [48]. In C2C12 muscle cells, ectopic expression of *FOXO1* markedly potentiates *PDK4* promoter activity. At the same time, activation of FOXO1 in the fasting state significantly upregulates *PDK4* expression, promotes fatty acid oxidation, reduces the inhibition of phosphodiesterase 4B phosphorylation, thereby increasing cAMP levels, activating the PKA-CREB signaling pathway, upregulating *G6Pase* and *PEPCK* expression, and potentiating gluconeogenesis [49–51]. In addition, in peripheral tissues including muscle, upregulation of *PDK4* expression inhibits pyruvate dehydrogenase activity, reduces the entry of pyruvate into the tricarboxylic acid cycle, facilitates the accumulation of pyruvate and its conversion into the gluconeogenic substrate lactate, and indirectly increases the body’s glucose output [52]. In summary, under the stress of overwintering starvation, AMPK and PGC-1α function cooperatively through multiple mechanisms to upregulate the expression of *FOXO1* and *PDK4*, promote the transcription of gluconeogenesis-related genes such as *PEPCK* and *G6Pase*, precisely regulate skeletal muscle gluconeogenesis, and at the same time reduce the consumption of the glucose precursor lactate, ensuring that skeletal muscle can maintain glucose synthesis and meet the body’s energy needs under energy-deficient conditions (Figure 1).

## ADIPOSE-SKELETAL MUSCLE METABOLIC CROSSTALK

Under winter starvation stress, AMPK and PGC-1α precisely regulate the coordination of energy metabolism between adipose and skeletal muscle to maintain the body’s energy metabolism homeostasis and normal physiological functions in short-term energy demand and long-term adaptive regulation. When energy supply is insufficient, AMPK phosphorylates the Ser79 site of ACC, inhibiting its activity, reducing the expression level of malonyl-CoA, relieving the inhibition of CPT1, and promoting the entry of fatty acids into mitochondria for beta-oxidation, thereby supplying essential energy substrates to skeletal muscle [53,54]. Studies have shown that cold stimulation promotes AMPK activation in lambs, phosphorylates ACC, reduces its activity, reduces the synthesis of malonyl-CoA, and enhances the activity of HSL and adipose triglyceride lipase, catalyzing the decomposition of triglycerides into free fatty acids; cold stimulation also promotes the release of norepinephrine from the SNS, activates β3-adrenergic receptors, activates the cAMP-PKA pathway, phosphorylates HSL, and synergistically facilitates the production of gluconeogenic precursor glycerol [55]. Under cold exposure, in skeletal muscle, the activation of the AMPK signaling pathway not only alleviates inhibition of CPT1 by phosphorylating and inhibiting ACC, reducing the level of malonyl-CoA, but also activates the transcriptional coactivator PGC-1α by phosphorylation and enhances the activity of the transcription factor PPARα, thereby significantly upregulating the expression level of the fatty acid oxidation-related gene *CPT1*, promoting fatty acid oxidation and enhancing the body’s energy supply [56]. Under cold exposure, the level of phosphorylated AMPKα protein in rat skeletal muscle increased significantly, upregulated the expression of *PGC-1α* and *PPARα*, inhibited ACC activity, and reduced the level of malonyl-CoA, thereby relieving the inhibition of CPT1, promoting the entry of fatty acids into mitochondria for oxidation, and potentiating the fatty acid oxidation capacity of skeletal muscle and the body’s energy supply efficiency [54]. AMPK/PGC-1α also upregulates the expression of lipoprotein receptor *cluster of differentiation 36* (*CD36*) and *fatty acid transport protein* (*FATP*), promoting the transport of lipolysis products to skeletal muscle and enhancing intramuscular gluconeogenesis under winter starvation. Studies have shown that AMPK reduces the m6A methylation modification of *Parkin* mRNA through FTO-dependent m6A demethylation, inhibits the binding of YTHDF2 protein to *Parkin* mRNA, reduces Parkin-mediated CD36 polyubiquitination, thereby increasing CD36 protein levels, and activates the AKT signaling pathway to promote the transport of CD36 to the plasma membrane in mouse muscles, enhance its membrane surface localization, and improve the efficiency of long-chain fatty acid uptake [57]. Similarly, in transgenic mice with muscle-specific overexpression of PGC-1α, PGC-1α functions primarily as a transcriptional coactivator and is recruited by transcription factors such as PPARα to the PPAR response element (PPRE) region on the promoters of genes including CD36 and FATP, thereby significantly upregulating the transcription levels of these FATPs and potentiating the transmembrane transport and oxidative utilization of fatty acids [58]. In addition, AMPK also potentiates the transcriptional activity of the transcription factor FOXO1 by phosphorylating it, thereby upregulating the expression of FATPs CD36 and FATP, promoting the transport of fatty acids, and supporting the oxidation of fatty acids in skeletal muscle. Studies have shown that fasting leads to a decrease in insulin levels and promotes the nuclear translocation and activation of FOXO1. Activated FOXO1 can significantly promote the translocation of CD36 from the intracellular to the plasma membrane and upregulate *ACOX* mRNA levels, thereby enhancing intracellular fatty acid uptake and peroxisomal fatty acid oxidation, and accelerating the uptake and utilization of fatty acids by skeletal muscle to meet energetic demands during fasting [59]. Therefore, under the stress of winter starvation, AMPK/PGC-1α inhibits ACC activity, upregulates CPT1 levels, and enhances the function of CD36 and FATP, thereby promoting the uptake and utilization of intramuscular fat by skeletal muscle, supplying animals with adequate energy and gluconeogenesis substrates, and maintaining energy homeostasis and life activities under low-temperature starvation.

## CONCLUSIONS AND FUTURE PERSPECTIVES

Under the stress of winter starvation, animals primarily activate the SNS-βAR-AMPK/PGC-1α pathway, upregulate the expression of *FOXO1* and *PDK4*, promote the transcription of gluconeogenesis-related genes including *PEPCK* and *G6Pase*, potentiate animal skeletal muscle gluconeogenesis, and reduce lactate metabolism, so that more metabolites flow to the gluconeogenesis pathway to meet the glucose demand of animal skeletal muscle. During this process, AMPK/PGC-1α is bidirectionally regulated by non-coding RNAs including miRNA, lncRNA, and circRNA to maintain the normal metabolic response of skeletal muscle under low temperature and starvation conditions. In addition, AMPK/PGC-1α promotes the uptake and utilization of the gluconeogenic precursor glycerol by skeletal muscle by inhibiting ACC activity and upregulating the expression of *UCP1*, *CPT1*, *FATP*, and *CD36*, thereby sustaining its metabolic demands during winter starvation. This review also explores the differences in energy metabolism between monogastric animals and ruminants by comparing their adaptive strategies in this process. Current research shows that exploring the mechanism of skeletal muscle gluconeogenesis under winter starvation stress plays an important role in promoting the breeding and improvement of cold-tolerant breeds in high-altitude areas and improving the production performance of livestock and poultry. However, research on the mechanism of gluconeogenesis in skeletal muscle of animals under winter starvation stress is still limited, and the regulation details of AMPK/PGC-1α pathway under this process is unknow. The differences of the process of gluconeogenesis in muscle and liver must still be unraveled. The adaptive mechanism of gluconeogenesis in different evolutionary lineages needs further exploration.

## Data Availability

Upon reasonable request, the datasets of this study can be available from the corresponding author.
